# Irritability and Social Media Use in US Adults

**DOI:** 10.1001/jamanetworkopen.2024.52807

**Published:** 2025-01-08

**Authors:** Roy H. Perlis, Ata Uslu, Jonathan Schulman, Faith M. Gunning, Mauricio Santillana, Matthew A. Baum, James N. Druckman, Katherine Ognyanova, David Lazer

**Affiliations:** 1 Center for Quantitative Health, Massachusetts General Hospital, Boston; 2 Department of Psychiatry, Harvard Medical School, Boston, Massachusetts; 3 Network Science Institute, Northeastern University, Boston, Massachusetts; 4 Institute for Quantitative Social Science, Harvard University, Boston, Massachusetts; 5 Department of Political Science, Northwestern University, Chicago, Illinois; 6 Weill Cornell Medicine, New York, New York; 7 John F. Kennedy School of Government and Department of Government, Harvard University, Cambridge, Massachusetts; 8 Department of Political Science, University of Rochester, Rochester, New York; 9 Department of Communication, School of Communication and Information, Rutgers University, New Brunswick, New Jersey

## Abstract

**Importance:**

Efforts to understand the complex association between social media use and mental health have focused on depression, with little investigation of other forms of negative affect, such as irritability and anxiety.

**Objective:**

To characterize the association between self-reported use of individual social media platforms and irritability among US adults.

**Design, Setting, and Participants:**

This survey study analyzed data from 2 waves of the COVID States Project, a nonprobability web-based survey conducted between November 2, 2023, and January 8, 2024, and applied multiple linear regression models to estimate associations with irritability. Survey respondents were aged 18 years and older.

**Exposure:**

Self-reported social media use.

**Main Outcomes and Measures:**

The primary outcome was score on the Brief Irritability Test (range, 5-30), with higher scores indicating greater irritability.

**Results:**

Across the 2 survey waves, there were 42 597 unique participants, with mean (SD) age 46.0 (17.0) years; 24 919 (58.5%) identified as women, 17 222 (40.4%) as men, and 456 (1.1%) as nonbinary. In the full sample, 1216 (2.9%) identified as Asian American, 5939 (13.9%) as Black, 5322 (12.5%) as Hispanic, 624 (1.5%) as Native American, 515 (1.2%) as Pacific Islander, 28 354 (66.6%) as White, and 627 (1.5%) as other (ie, selecting the other option prompted the opportunity to provide a free-text self-description). In total, 33 325 (78.2%) of the survey respondents reported daily use of at least 1 social media platform, including 6037 (14.2%) using once a day, 16 678 (39.2%) using multiple times a day, and 10 610 (24.9%) using most of the day. Frequent use of social media was associated with significantly greater irritability in univariate regression models (for more than once a day vs never, 1.43 points [95% CI, 1.22-1.63 points]; for most of the day vs never, 3.37 points [95% CI, 3.15-3.60 points]) and adjusted models (for more than once a day, 0.38 points [95% CI, 0.18-0.58 points]; for most of the day, 1.55 points [95% CI, 1.32-1.78 points]). These associations persisted after incorporating measures of political engagement.

**Conclusions and Relevance:**

In this survey study of 42 597 US adults, irritability represented another correlate of social media use that merits further characterization, in light of known associations with depression and suicidality.

## Key Points

**Question:**

Is social media use by adults associated with irritability, or being prone to anger?

**Findings:**

In this survey study of 42 597 US adults, high levels of social media use, in particular frequent posting, were associated with greater irritability in cross-sectional analysis.

**Meaning:**

The association between social media and irritability merits further attention, given the known associations between irritability and adverse outcomes.

## Teaser

This survey study examines the association between self-reported use of individual social media platforms and irritability among US adults.

## Introduction

If you’re not outraged, you’re not paying attention.

**Anonymous** ^1^

The association between social media use and depressive symptoms has been documented in adolescents,^2^ young adults,^3^ and subsequently in adults across the lifespan.^4^ Untangling causation in this association has proven to be challenging, because most studies rely on cross-sectional data. The sole randomized clinical trial^5^ suggested that discontinuation of Facebook use was associated with improved mood; other longitudinal studies^4,6,7^ suggest that the association may be complex and bidirectional.

In their focus on depressive symptoms, such studies have tended to neglect other forms of negative affect, most notably irritability, or being prone to anger. A more precise understanding of the range of affect associated with social media use could facilitate efforts to mitigate such symptoms. Furthermore, little is known about whether specific content on social media contributes to negative affect, in particular, whether political engagement may explain some of the observed associations. To address these gaps, we drew on 2 waves of an internet survey^8^ conducted in all 50 US states and the District of Columbia. Using quotas and weighting to achieve a representative population of US adults, the survey assessed both frequency of social media use and current irritability, as well as other negative affective symptoms.

## Methods

### Study Design

In this survey study, we used data from 2 waves of an internet survey^8^ conducted between November 2, 2023, and January 8, 2024, by a multipanel commercial vendor, PureSpectrum. We applied a nonprobability sampling design, with state-level quotas to ensure representativeness of gender, age, and race and ethnicity within each state. The survey applied attention checks and open-ended questions to filter out unreliable or automated respondents. This study was formally reviewed and approved by the institutional review board of Harvard University as exempt because only deidentified data were used and no participant contact was required. Respondents provided informed consent online before answering survey questions. We followed American Association for Public Opinion Research (AAPOR) reporting guidelines for reporting survey results.

### Measures

#### Sociodemographic Features

Sociodemographic features were collected via self-report and included age, gender, education, employment status, and race and ethnicity. The latter 2 categories could be selected from a list including African American or Black, Asian American, Hispanic, Native American, Pacific Islander, White, or other (ie, selecting the other option prompted the opportunity to provide a free-text self-description). Race and ethnicity were collected to confirm representativeness of the US population and are reported as advised in a recent guidance statement.^9^ As in prior work, to facilitate the inclusion of smaller groups, we collapsed individuals identifying as Native American, Asian, Pacific Islander, and other into a single category for analysis, and dichotomized employment to working full-time vs all others.

#### Social Media Use

Social media use was collected by asking, “Do you ever use any of the following social media sites or apps?” Respondents who answered affirmatively were then asked to indicate their frequency of use for each (less than once a week, once a week, several times a week, about once a day, several times a day, or most of the day), and frequency of posting (never, less than once a month, about once a month, about once a week, about once a day, or multiple times a day). A value for maximum frequency of use, and maximum frequency of posting, across platforms was also derived by taking the value for the platform with maximal use. For the present study, we analyzed Instagram, Twitter/X, Facebook, and TikTok use.

#### Political Engagement

Political engagement was assessed using 2 items. The first asked, “How closely do you follow news and information about politics and current affairs?” with choices including not closely at all, not very closely, somewhat closely, or very closely. The second asked, “How often do you talk to people about politics and current affairs, either in person, over the phone, or electronically?” with response options of never, less than once a week, once a week, a few times a week, daily, or a few times a day. Respondents were also asked to identify their political affiliation (Republican, Democrat, Independent, or other); for purposes of analysis, Independent and other were pooled.

#### Negative Affect

Negative affect was measured using 3 scales validated for use as screening or outpatient measures. Irritability was measured via the Brief Irritability Test (BITe5), which incorporates 5 statements assessing the prior 2 weeks beginning with, “Please indicate how often you have felt or behaved in the following ways, during the past two weeks, including today.”^10^ These 5 statements include, “I have been grumpy,” “I have been feeling like I might snap,” “Other people have been getting on my nerves,” “Things have been bothering me more than they normally do,” and “I have been feeling irritable.” Frequency is scored between 1 and 6, from never to always, with individual items summed to yield a total score between 5 and 30, with higher scores indicating greater irritability. The BITe5 was previously shown to exhibit minimal gender effects, strong internal consistency, and minimal overlap with depression or anger.^10^ We assessed depressive symptom severity with the 9-item Patient Health Questionnaire,^11,12^ which incorporates the diagnostic criteria for major depressive disorder in the *Diagnostic and Statistical Manual of Mental Disorders* (Fifth Edition) scored on a Likert scale from 0 (not at all) to 3 (nearly every day). A 10 or greater on this measure is considered to be at least moderate depression. We measured anxiety with the 2-item Generalized Anxiety Disorder screen,^13^ with the same anchor points; a 3 or greater on this measure is considered a positive screen for generalized anxiety.

### Statistical Analysis

All analyses used R statistical software version 4.3.2^14^ and the R survey package version 4.2-1^15^ (both from R Project for Statistical Computing). We used unweighted results to generate the cohort description shown in the Table; all other results applied survey weights to approximate national distributions. All *P* values reported are from 2-sided tests with statistical significance set at *P* < .05. All adjusted linear regression models included the following features: age category, gender, education (graduate, undergraduate, some college, high school graduate, or some high school or less), annual household income (<$25 000, $25 000 to <$50 000, $50 000 to <$100 000, and ≥$100 000), race and ethnicity, and rural, suburban, or urban setting. Extended linear regression models also included anxiety, measured by the 2-item Generalized Anxiety Disorder screen, and depressive symptoms, measured by the 9-item Patient Health Questionnaire, as a means of understanding the extent to which social media use was associated with irritability symptoms beyond those explained by anxiety and depression. Finally, we incorporated measures of political engagement in linear regression models, to consider the possibility that such engagement could either confound, or mediate, any observed associations.

**Table.** Full Cohort Demographics and Frequency of Social Media Use

Characteristic	Participants, No. (%)			*P* value
	No daily use (n = 9272)	Daily use (n = 33 325)	Total (N = 42 597)	
Age, mean (SD), y	52.7 (17.3)	44.1 (16.4)	46.0 (17.0)	<.001
Gender				
Female	4445 (47.9)	20 474 (61.4)	24 919 (58.5)	<.001
Male	4719 (50.9)	12 503 (37.5)	17 222 (40.4)	
Nonbinary	108 (1.2)	348 (1.0)	456 (1.1)	
Race and ethnicity				
Asian American	261 (2.8)	955 (2.9)	1216 (2.9)	<.001
Black	1172 (12.6)	4767 (14.3)	5939 (13.9)	
Hispanic	876 (9.4)	4446 (13.3)	5322 (12.5)	
Native American	126 (1.4)	498 (1.5)	624 (1.5)	
Pacific Islander	112 (1.2)	403 (1.2)	515 (1.2)	
White	6543 (70.6)	21 811 (65.4)	28 354 (66.6)	
Other^a^	182 (2.0)	445 (1.3)	627 (1.5)	
Education				
Some high school or less	332 (3.6)	1316 (3.9)	1648 (3.9)	<.001
High school graduate	2041 (22.0)	8315 (25.0)	10 356 (24.3)	
Some college	2343 (25.3)	8835 (26.5)	11 178 (26.2)	
College degree	3307 (35.7)	11 179 (33.5)	14 486 (34.0)	
Graduate degree	1249 (13.5)	3680 (11.0)	4929 (11.6)	
Employment				
Full time	2886 (31.1)	13 888 (41.7)	16 774 (39.4)	<.001
Gig or contract	93 (1.0)	280 (0.8)	373 (0.9)	
Homemaker	448 (4.8)	2036 (6.1)	2484 (5.8)	
Part time	850 (9.2)	3756 (11.3)	4606 (10.8)	
Retired	2854 (30.8)	5644 (16.9)	8498 (19.9)	
Self-employed	708 (7.6)	2427 (7.3)	3135 (7.4)	
Student	250 (2.7)	1495 (4.5)	1745 (4.1)	
Unemployed	1183 (12.8)	3799 (11.4)	4982 (11.7)	
Annual household income, $				
<25 000	2145 (23.1)	7342 (22.0)	9487 (22.3)	.008
25 000 to <50 000	2600 (28.0)	9049 (27.2)	11 649 (27.3)	
50 000 to <100 000	2909 (31.4)	10 887 (32.7)	13 796 (32.4)	
≥100 000	1618 (17.5)	6047 (18.1)	7665 (18.0)	
Total social media use				
Never	1520 (16.4)	6028 (18.1)	7548 (17.7)	<.001
Less than once per week	5439 (58.7)	18 829 (56.5)	24 268 (57.0)	
Once per week	2313 (24.9)	8468 (25.4)	10 781 (25.3)	
Several times per week				
Once per day	12.5 (5.3)	13.9 (5.6)	13.6 (5.6)	<.001
Several times per day	0	16 678 (50.0)	16 678 (39.2)	
Most of the day	0	10 610 (31.8)	10 610 (24.9)	

^a^ Selecting the other option prompted the opportunity to provide a free-text self-description.

Rates of missing data were extremely low, so we did not apply multiple imputation. Because participants could return for the subsequent survey wave, we elected a priori to include in the primary analysis only the initial (index) survey completed. In prior work^16^ using earlier survey waves, randomly selecting a wave or including multiple observations per individual yielded very similar results.

## Results

Across the 2 survey waves, there were 42 597 unique participants (mean [SD] age, 46.0 [17.0] years; 24 919 [58.5%] identified as women; 17 222 [40.4%] identified as men; 456 [1.1%] identified as nonbinary). In the full sample, 1216 (2.9%) identified as Asian American, 5939 (13.9%) as Black, 5322 (12.5%) as Hispanic, 624 (1.5%) as Native American, 515 (1.2%) as Pacific Islander, 28 354 (66.6%) as White, and 627 (1.5%) as other. Additional characteristics of the survey cohort are summarized in Table.

**Figure 1.**
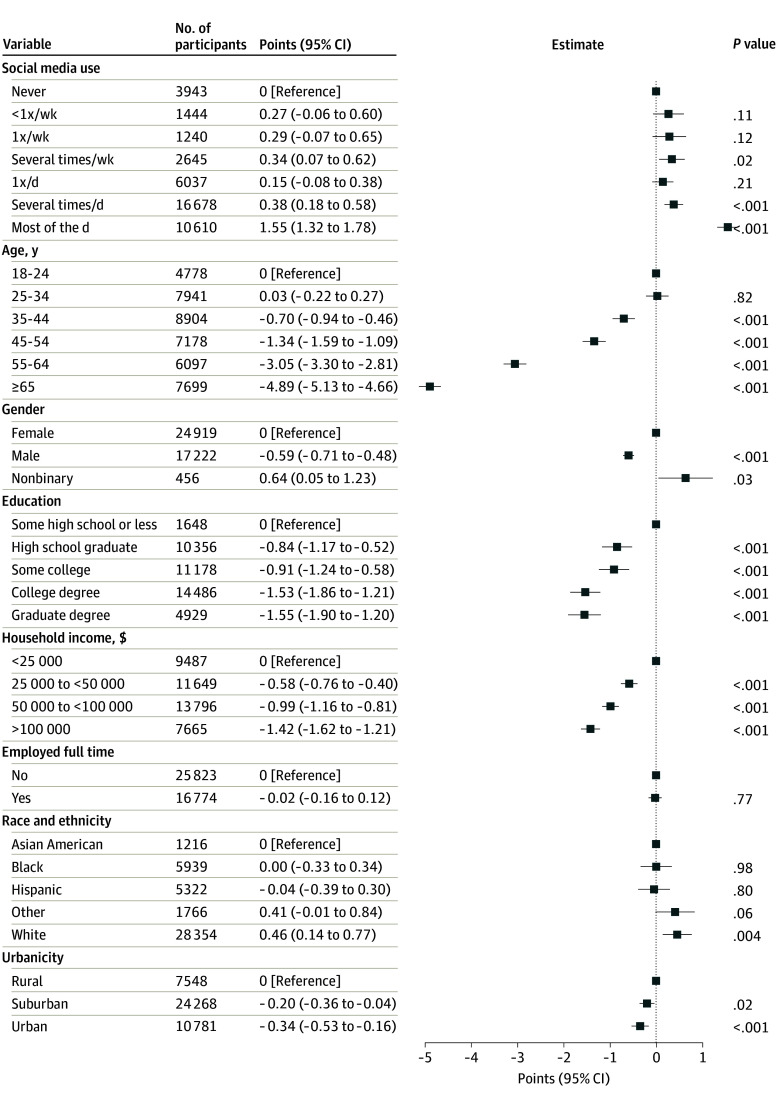
Adjusted Associations Between Maximal Amount of Social Media Usage and Degree of Irritability Irritability was measured via the Brief Irritability Test (score range, 5-30, with higher scores indicating greater irritability).

In total, 33 325 of the 42 597 survey respondents (78.2%) reported daily use of at least 1 social media platform, including 6037 (14.2%) using once a day, 16 678 (39.2%) using multiple times a day, and 10 610 (24.9%) using most of the day (Figure 1). eTable 1 in Supplement 1 reports utilization by individual platform. Asked to report their frequency of posting on social media, 7827 respondents (18.4%) reported posting at least weekly, 5822 (13.7%) at least daily, and 6905 (16.2%) multiple times per day (eTable 2 in Supplement 1).

We first examined the association between maximal amount of social media use and degree of irritability (Figure 1). For the former, use more than once a day was associated with significantly greater irritability score in univariate linear regression models (for more than once a day, an increase in BITe5 score of 1.43 points [95% CI, 1.22-1.63 points]; for most of the day, 3.37 points [95% CI, 3.15-3.60 points]) and adjusted models (for more than once a day, 0.38 points [95% CI, 0.18-0.58 points]; for most of the day, 1.55 points [95% CI, 1.32-1.78 points]). Statistically significant but attenuated associations persisted after inclusion of depression and anxiety severity in regression models—that is, more modest increases in irritability were observed after accounting for that explained by depressive and anxious symptoms (eFigure 1 in Supplement 1).

**Figure 2.**
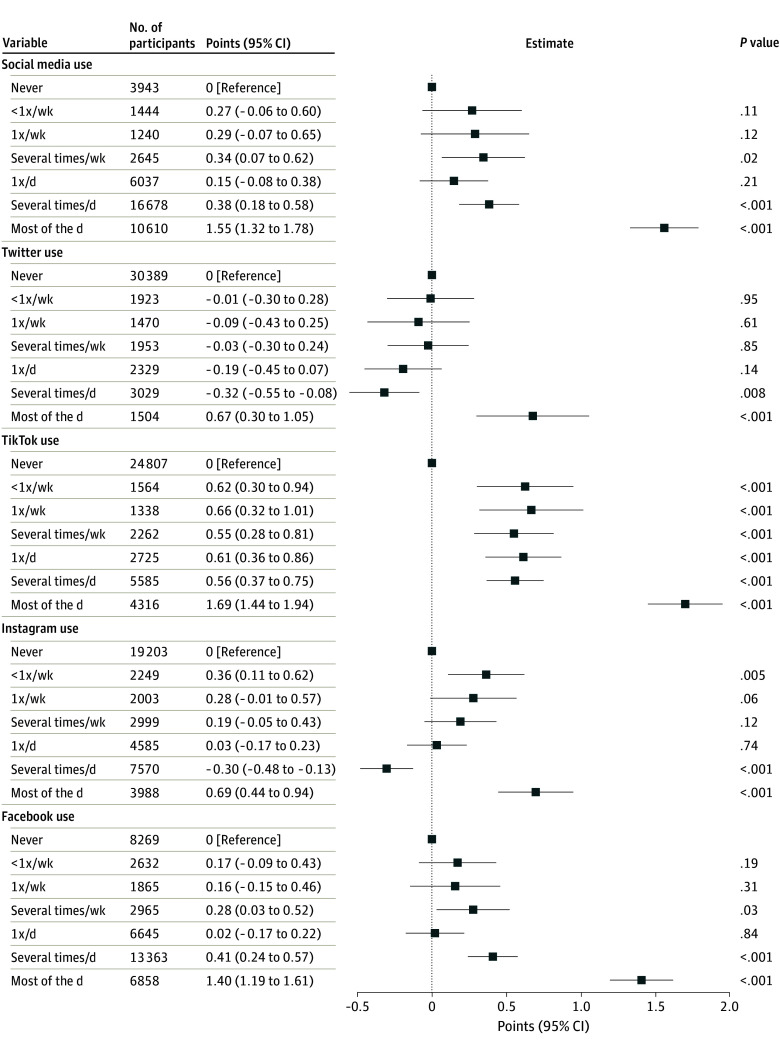
Adjusted Associations Between Daily Social Media Use and Irritability Irritability was measured via the Brief Irritability Test (score range, 5-30, with higher scores indicating greater irritability).

We then examined these associations for individual social media platforms, focusing on Instagram, Twitter, Facebook, and TikTok. Figure 2 shows the association between daily use and irritability for each of these in adjusted models. For use most of the day, significant increases in irritability were identified for Twitter (0.67 points; 95% CI, 0.30-1.05 points), TikTok (1.69 points; 95% CI, 1.44-1.94 points), Instagram (0.69 points; 95% CI, 0.44-0.94 points), and Facebook (1.40 points; 95% CI, 1.19-1.61 points); more modest increases were associated with use several times per day for all except Twitter and Instagram. Corresponding results from regression models also incorporating depressive and anxious symptoms are illustrated in eFigure 2 in Supplement 1; as with the analyses of overall social media use in eFigure 1 in Supplement 1, they indicate that some, but not all, of the increase in irritability is accounted for by increase in depressive and anxious symptoms.

**Figure 3.**
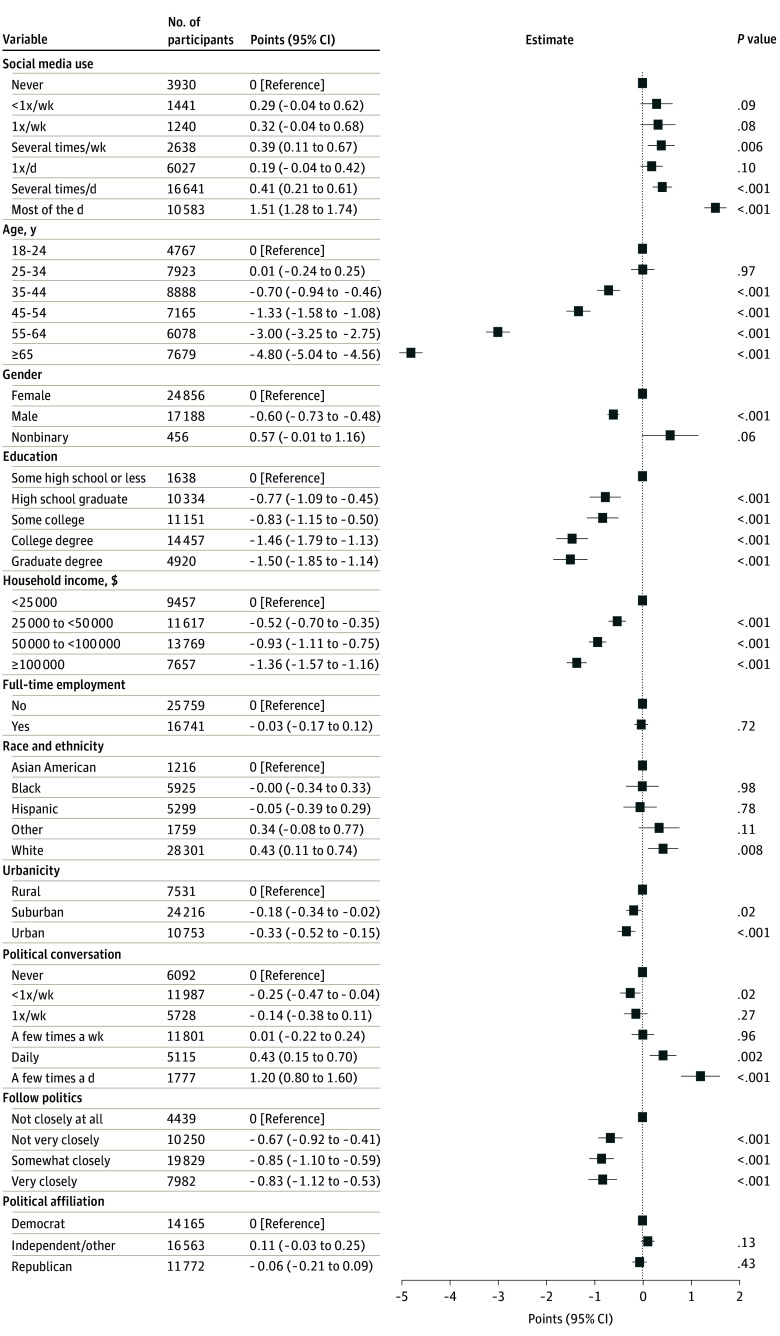
Adjusted Associations Between Social Media Use and Irritability Including Terms for Frequency of Political Discussion Irritability was measured via the Brief Irritability Test (score range, 5-30, with higher scores indicating greater irritability).

We next sought to understand whether interest in and exposure to political views was associated with irritability, fitting linear regression models that included these additional terms. Figure 3 shows adjusted associations between social media use and irritability, including terms for frequency of political discussion, how closely individuals follow political information, and political affiliation. More frequent political discussions were associated with greater irritability (for discussion a few times a day, 1.20 points; 95% CI, 0.80-1.60 points); following political news even not very closely was associated with modest decreases in irritability. No significant difference was observed for political affiliation. In models incorporating these political variables, social media use remained associated with greater irritability for use several times a day (0.41 points; 95% CI, 0.21-0.61 points) and most of the day (1.51 points; 95% CI, 1.28-1.74 points).

**Figure 4.**
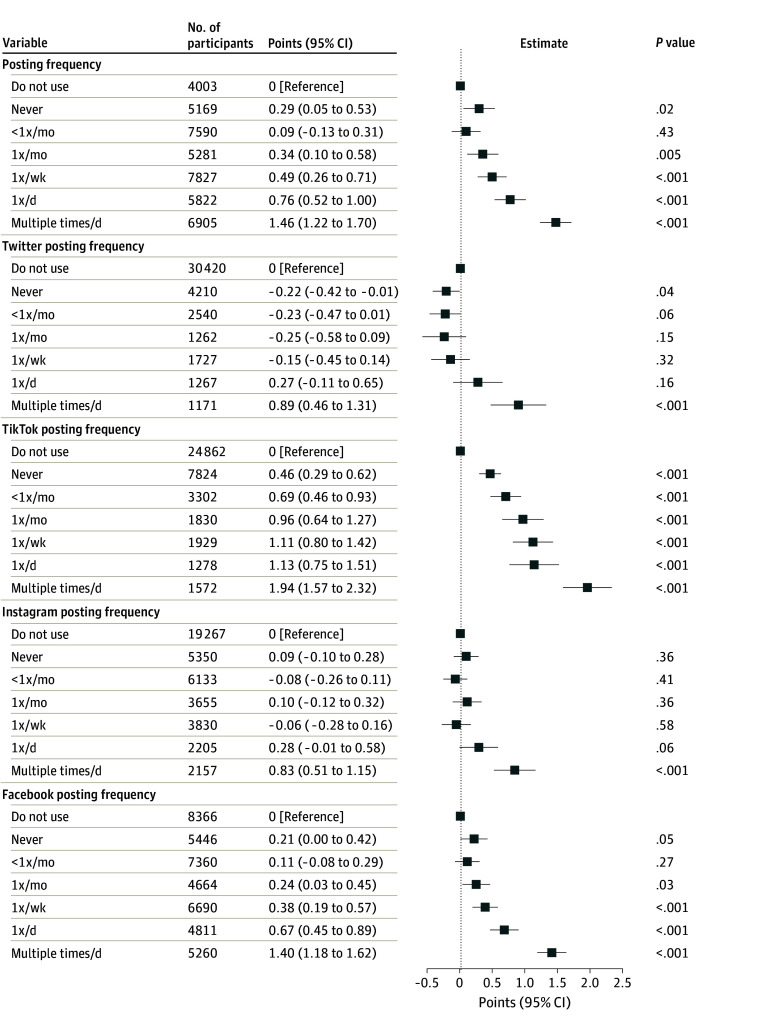
Adjusted Associations Between Social Media Use and Irritability for Each Social Media Platform by Posting Frequency Irritability was measured via the Brief Irritability Test (score range, 5-30, with higher scores indicating greater irritability).

Finally, we examined whether more active engagement with social media, in terms of frequency of posting rather than just using the platform, was associated with greater levels of irritability. Figure 4 displays adjusted coefficients for each platform and frequency of posting, compared with nonuse. For each platform, we identified a dose-response association, with greater frequency of posting associated with greater levels of irritability.

## Discussion

In this survey study of more than 42 500 US adults in all 50 US states and the District of Columbia, we found that frequent users of social media experienced increased levels of irritability, above and beyond that explained by depression or anxiety. A dose-response pattern was particularly apparent when frequency of posting (ie, active rather than passive engagement) was considered, although the particular pattern and magnitude varied by platform.

We hypothesized that political engagement might confound the association between social media and irritability (ie, that greater utilization might simply reflect greater political interest or more frequent conversations). However, incorporating terms for these measures, as well as political affiliation, did not meaningfully change associations between social media use and irritability for the survey population as a whole.

In prior work,^4^ we identified an association between social media use and depressive symptoms, either cross-sectionally or at a subsequent survey. Earlier cross-sectional studies identified increased depressive symptoms in adolescents^2^ or young adults^3^ using social media, as did short-term longitudinal studies.^6,7^ A randomized Facebook discontinuation study^5^ identified corresponding improvements in mood following discontinuation.

Irritability is often considered simply a correlate of other forms of negative valence, most notably depression or anxiety. However, when it co-occurs with depression, it may be associated with greater functional impact,^17,18^ poorer treatment outcomes,^19^ and likelihood of suicidal thoughts and behaviors.^8,20,21^ Irritability has also been associated with impacts on social function and employment, as well as risk for violence.^22^ The possibility that social media use may contribute to irritability, or at least that it has a bidirectional association with irritability, is therefore of more than academic interest.

Our work also complements a substantial body of work examining affect as it relates to political activity. The bulk of this prior work relates to anger,^23^ anxiety,^24^ or the combination thereof.^25^ Our findings suggest the critical nature of considering irritability as an important form of negative valence in its own right. As such, the observation that high levels of social media use correlates with irritability may have further real-world consequences that merit further study.

### Limitations

This study has multiple limitations. Most importantly, we cannot assess causation in light of the cross-sectional design we use. The association between social media and mood is likely to be complex and potentially bidirectional. For example, it has been suggested that some social media platforms and algorithms actually seek to elicit outrage as a means of increasing engagement.^26^ We could not analyze specific social media content for this cohort, so we cannot relate the irritability to a specific domain.^27^ Furthermore, because we rely on self-report rather than objective measures, we cannot exclude recall bias or other forms of confounding.

## Conclusions

Our results suggest an association between high levels of social media use, particularly posting on social media, and irritability among US adults. The implications of this irritability and the potential for interventions to address this association require additional investigation.

## Notes

Supplement 1. **eTable 1.** Frequency of social media use, full cohort **eTable 2.** Frequency of posting on social media, full cohort **eFigure 1.** Linear regression model of irritability, adjusted for depressive and anxious symptoms **eFigure 2.** Adjusted associations between frequency of social media use and irritability, with inclusion of depressive and anxious symptoms

Supplement 2.
Data Sharing Statement
