# Supplementary material for: Irritability and Social Media Use in US Adults
Source: JAMA Netw Open. 2025 Jan 8;8(1):e2452807. doi: 10.1001/jamanetworkopen.2024.52807 (PMC12527479; doi:10.1001/jamanetworkopen.2024.52807)
Supplement: Supplement 1. — eTable 1. Frequency of social media use, full cohort eTable 2. Frequency of posting on social media, full cohort eFigure 1. Linear regression model of irritability, adjusted for depressive and anxious symptoms eFigure 2. Adjusted associations between frequency of social media use and irritability, with inclusion of depressive and anxious symptoms [file jamanetwopen-e2452807-s001.pdf]

Supplemental Online Content

Perlis RH, Uslu A, Schulman J, et al. Irritability and social media use in US adults. *JAMA Netw Open*. 2025;8(1):e2452807. doi:10.1001/jamanetworkopen.2024.52807

eTable 1. Frequency of social media use, full cohort
eTable 2. Frequency of posting on social media, full cohort
eFigure 1. Linear regression model of irritability, adjusted for depressive and anxious symptoms
eFigure 2. Adjusted associations between frequency of social media use and irritability, with inclusion of depressive and anxious symptoms

This supplemental material has been provided by the authors to give readers additional information about their work.

© 20245 Perlis RH et al. *JAMA Network Open*.

eTable 1. Frequency of social media use, full cohort

	No daily social media (N=9272)	Daily social media (N = 33325)	Total (N=42597)	p value
**Total social media**				< 0.001
never	3943 (42.5%)	0 (0.0%)	3943 (9.3%)	
<1x/week	1444 (15.6%)	0 (0.0%)	1444 (3.4%)	
1x/week	1240 (13.4%)	0 (0.0%)	1240 (2.9%)	
several	2645 (28.5%)	0 (0.0%)	2645 (6.2%)	
1x/day	0 (0.0%)	6037 (18.1%)	6037 (14.2%)	
several times/day	0 (0.0%)	16678 (50.0%)	16678 (39.2%)	
most of the day	0 (0.0%)	10610 (31.8%)	10610 (24.9%)	
**Twitter use**				< 0.001
never	8081 (87.2%)	22308 (66.9%)	30389 (71.3%)	
<1x/week	468 (5.0%)	1455 (4.4%)	1923 (4.5%)	
1x/week	321 (3.5%)	1149 (3.4%)	1470 (3.5%)	
several	402 (4.3%)	1551 (4.7%)	1953 (4.6%)	
1x/day	0 (0.0%)	2329 (7.0%)	2329 (5.5%)	
several times/day	0 (0.0%)	3029 (9.1%)	3029 (7.1%)	
most of the day	0 (0.0%)	1504 (4.5%)	1504 (3.5%)	
**Tiktok use**				< 0.001
never	8003 (86.3%)	16804 (50.4%)	24807 (58.2%)	
<1x/week	446 (4.8%)	1118 (3.4%)	1564 (3.7%)	
1x/week	285 (3.1%)	1053 (3.2%)	1338 (3.1%)	
several	538 (5.8%)	1724 (5.2%)	2262 (5.3%)	
1x/day	0 (0.0%)	2725 (8.2%)	2725 (6.4%)	
several times/day	0 (0.0%)	5585 (16.8%)	5585 (13.1%)	
most of the day	0 (0.0%)	4316 (13.0%)	4316 (10.1%)	
**Instagram use**				< 0.001
never	7266 (78.4%)	11937 (35.8%)	19203 (45.1%)	
<1x/week	743 (8.0%)	1506 (4.5%)	2249 (5.3%)	
1x/week	497 (5.4%)	1506 (4.5%)	2003 (4.7%)	
several	766 (8.3%)	2233 (6.7%)	2999 (7.0%)	
1x/day	0 (0.0%)	4585 (13.8%)	4585 (10.8%)	
several times/day	0 (0.0%)	7570 (22.7%)	7570 (17.8%)	
most of the day	0 (0.0%)	3988 (12.0%)	3988 (9.4%)	
**Facebook use**				< 0.001
never	4823 (52.0%)	3446 (10.3%)	8269 (19.4%)	
<1x/week	1499 (16.2%)	1133 (3.4%)	2632 (6.2%)	
1x/week	1061 (11.4%)	804 (2.4%)	1865 (4.4%)	
several	1889 (20.4%)	1076 (3.2%)	2965 (7.0%)	
1x/day	0 (0.0%)	6645 (19.9%)	6645 (15.6%)	
several times/day	0 (0.0%)	13363 (40.1%)	13363 (31.4%)	
most of the day	0 (0.0%)	6858 (20.6%)	6858 (16.1%)	

© 2025 Perlis RH et al. *JAMA Network Open.*

eTable 2. Frequency of posting on social media, full cohort

	No daily social media (N=9272)	Daily social media (N=33325)	Total (N=42597)	p value
**Total posting**				< 0.001
do not use	3958 (42.7%)	45 (0.1%)	4003 (9.4%)	
never	1867 (20.1%)	3302 (9.9%)	5169 (12.1%)	
<1x/month	1845 (19.9%)	5745 (17.2%)	7590 (17.8%)	
1x/month	694 (7.5%)	4587 (13.8%)	5281 (12.4%)	
1x/week	699 (7.5%)	7128 (21.4%)	7827 (18.4%)	
1x/day	141 (1.5%)	5681 (17.0%)	5822 (13.7%)	
multiple times/day	68 (0.7%)	6837 (20.5%)	6905 (16.2%)	
**Twitter posting**				< 0.001
do not use	8083 (87.2%)	22337 (67.0%)	30420 (71.4%)	
never	571 (6.2%)	3639 (10.9%)	4210 (9.9%)	
<1x/month	354 (3.8%)	2186 (6.6%)	2540 (6.0%)	
1x/month	117 (1.3%)	1145 (3.4%)	1262 (3.0%)	
1x/week	123 (1.3%)	1604 (4.8%)	1727 (4.1%)	
1x/day	20 (0.2%)	1247 (3.7%)	1267 (3.0%)	
multiple times/day	4 (0.0%)	1167 (3.5%)	1171 (2.7%)	
**Tiktok posting**				< 0.001
do not use	8012 (86.4%)	16850 (50.6%)	24862 (58.4%)	
never	820 (8.8%)	7004 (21.0%)	7824 (18.4%)	
<1x/month	254 (2.7%)	3048 (9.1%)	3302 (7.8%)	
1x/month	68 (0.7%)	1762 (5.3%)	1830 (4.3%)	
1x/week	76 (0.8%)	1853 (5.6%)	1929 (4.5%)	
1x/day	30 (0.3%)	1248 (3.7%)	1278 (3.0%)	
multiple times/day	12 (0.1%)	1560 (4.7%)	1572 (3.7%)	
**Instagram posting**				< 0.001
do not use	7269 (78.4%)	11998 (36.0%)	19267 (45.2%)	
never	901 (9.7%)	4449 (13.4%)	5350 (12.6%)	
<1x/month	644 (6.9%)	5489 (16.5%)	6133 (14.4%)	
1x/month	222 (2.4%)	3433 (10.3%)	3655 (8.6%)	
1x/week	190 (2.0%)	3640 (10.9%)	3830 (9.0%)	
1x/day	33 (0.4%)	2172 (6.5%)	2205 (5.2%)	
multiple times/day	13 (0.1%)	2144 (6.4%)	2157 (5.1%)	
**Facebook posting**				< 0.001
do not use	4839 (52.2%)	3527 (10.6%)	8366 (19.6%)	
never	1565 (16.9%)	3881 (11.6%)	5446 (12.8%)	
<1x/month	1596 (17.2%)	5764 (17.3%)	7360 (17.3%)	
1x/month	579 (6.2%)	4085 (12.3%)	4664 (10.9%)	
1x/week	543 (5.9%)	6147 (18.4%)	6690 (15.7%)	
1x/day	97 (1.0%)	4714 (14.1%)	4811 (11.3%)	
multiple times/day	53 (0.6%)	5207 (15.6%)	5260 (12.3%)	

© 2025 Perlis RH et al. *JAMA Network Open*.

eFigure 1. Linear regression model of irritability, adjusted for depressive and anxious symptoms

Variable		N	Estimate	p
**PHQ9 total**		42597	0.36 (0.35, 0.38)	<0.001
**GAD2 total**		42597	0.85 (0.80, 0.89)	<0.001
**Social media use**	never	3943	Reference	
	<1x/week	1444	−0.14 (−0.38, 0.10)	0.257
	1x/week	1240	−0.08 (−0.35, 0.18)	0.533
	several times/week	2645	0.06 (−0.14, 0.27)	0.543
	1x/day	6037	−0.08 (−0.24, 0.09)	0.369
	several times/day	16678	0.21 (0.06, 0.35)	0.006
	most of the day	10610	0.65 (0.49, 0.82)	<0.001
**Age**	18 to 24	4778	Reference	
	25 to 34	7941	0.06 (−0.12, 0.25)	0.494
	35 to 44	8904	0.02 (−0.16, 0.20)	0.833
	45 to 54	7178	−0.20 (−0.38, −0.01)	0.038
	55 to 64	6097	−0.79 (−0.98, −0.60)	<0.001
	65 and over	7699	−1.53 (−1.72, −1.35)	<0.001
**Gender**	Female	24919	Reference	
	Male	17222	−0.20 (−0.29, −0.11)	<0.001
	Nonbinary	456	−0.87 (−1.36, −0.37)	<0.001
**Education**	Some High School or Less	1648	Reference	
	High School Graduate	10356	−0.41 (−0.64, −0.17)	<0.001
	Some College	11178	−0.51 (−0.74, −0.27)	<0.001
	College Degree	14486	−0.67 (−0.91, −0.44)	<0.001
	Graduate Degree	4929	−0.68 (−0.94, −0.43)	<0.001
**Income**	<$25k	9487	Reference	
	$25k-<$50k	11649	−0.11 (−0.24, 0.02)	0.109
	$50k-<$100k	13796	−0.10 (−0.24, 0.03)	0.118
	$100k+	7665	−0.12 (−0.27, 0.03)	0.129
**Full time employment**	No	25823	Reference	
	Yes	16774	0.32 (0.22, 0.42)	<0.001
**Race and ethnicity**	Asian American	1216	Reference	
	Black	5939	0.21 (−0.04, 0.45)	0.097
	Hispanic	5322	0.01 (−0.23, 0.26)	0.916
	Other	1766	0.09 (−0.23, 0.41)	0.576
	White	28354	0.06 (−0.17, 0.28)	0.626
**Urbanicity**	Rural	7548	Reference	
	Suburban	24268	−0.12 (−0.24, −0.01)	0.037
	Urban	10781	−0.17 (−0.30, −0.03)	0.015

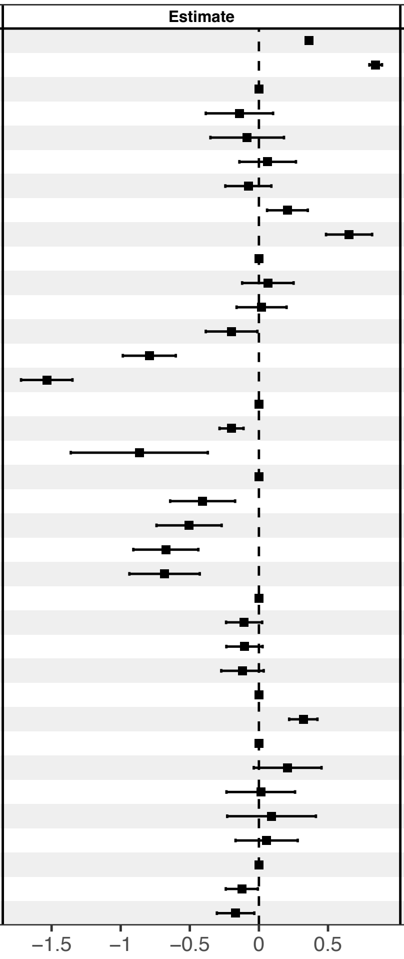

© 2025 Perlis RH et al. *JAMA Network Open*.

eFigure 2. Adjusted associations between frequency of social media use and irritability, with inclusion of depressive and anxious symptoms

Variable		N	Estimate	p
**Social media use**	never	3943	Reference	
	<1x/week	1444	−0.14 (−0.38, 0.10)	0.257
	1x/week	1240	−0.08 (−0.35, 0.18)	0.533
	several times/week	2645	0.06 (−0.14, 0.27)	0.543
	1x/day	6037	−0.08 (−0.24, 0.09)	0.369
	several times/day	16678	0.21 (0.06, 0.35)	0.006
	most of the day	10610	0.65 (0.49, 0.82)	<0.001
**Twitter use**	never	30389	Reference	
	<1x/week	1923	−0.22 (−0.43, −0.01)	0.043
	1x/week	1470	−0.23 (−0.47, 0.01)	0.057
	several times/week	1953	−0.13 (−0.33, 0.07)	0.196
	1x/day	2329	−0.21 (−0.40, −0.03)	0.026
	several times/day	3029	−0.27 (−0.44, −0.11)	0.001
	most of the day	1504	0.12 (−0.16, 0.39)	0.409
**Tiktok use**	never	24807	Reference	
	<1x/week	1564	0.01 (−0.23, 0.24)	0.950
	1x/week	1338	0.12 (−0.13, 0.37)	0.358
	several times/week	2262	0.15 (−0.04, 0.34)	0.116
	1x/day	2725	0.13 (−0.05, 0.32)	0.164
	several times/day	5585	0.26 (0.12, 0.41)	<0.001
	most of the day	4316	0.77 (0.59, 0.95)	<0.001
**Instagram use**	never	19203	Reference	
	<1x/week	2249	−0.05 (−0.23, 0.14)	0.624
	1x/week	2003	−0.11 (−0.31, 0.10)	0.322
	several times/week	2999	−0.05 (−0.23, 0.12)	0.555
	1x/day	4585	−0.06 (−0.21, 0.09)	0.414
	several times/day	7570	−0.07 (−0.20, 0.06)	0.296
	most of the day	3988	0.29 (0.11, 0.47)	0.002
**Facebook use**	never	8269	Reference	
	<1x/week	2632	−0.09 (−0.29, 0.10)	0.354
	1x/week	1865	0.01 (−0.21, 0.23)	0.933
	several times/week	2965	0.01 (−0.17, 0.20)	0.894
	1x/day	6645	0.03 (−0.11, 0.17)	0.675
	several times/day	13363	0.35 (0.23, 0.48)	<0.001
	most of the day	6858	0.72 (0.56, 0.87)	<0.001

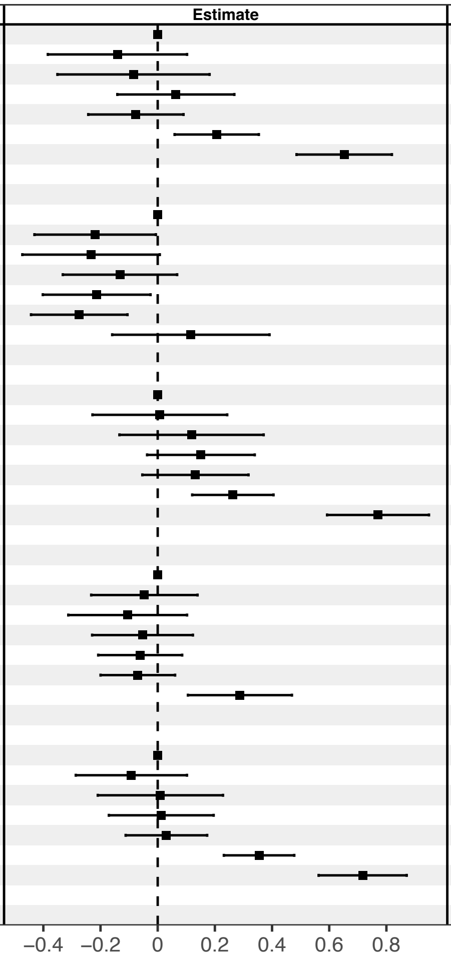

© 2025 Perlis RH et al. *JAMA Network Open*.
