# Supplementary material for: Irritability and Social Media Use in US Adults
Source: JAMA Netw Open. 2025 Jan 8;8(1):e2452807. doi: 10.1001/jamanetworkopen.2024.52807 (PMC12527479; doi:10.1001/jamanetworkopen.2024.52807)
Supplement: Supplement 2. — Data Sharing Statement [file jamanetwopen-e2452807-s002.pdf]

Data Sharing Statement

Perlis. Irritability and Social Media Use in US Adults. *JAMA Netw Open*. Published January 08, 2025. doi:10.1001/jamanetworkopen.2024.52807

Data
Data available: No
